# Prevalence, clinical outcomes and rainfall association of acute respiratory infection by human metapneumovirus in children in Bogotá, Colombia

**DOI:** 10.1186/s12887-019-1734-x

**Published:** 2019-10-10

**Authors:** Obando Evelyn, Fernández-Sarmiento Jaime, Montoya David, Acevedo Lorena, Arroyave Jenifer, Gamboa Oscar

**Affiliations:** 1 Department of Pediatrics and Intensive Care, Universidad de La Sabana, Fundación Cardioinfantil-Instituto de Cardiología, Bogotá, Colombia; 2 Department of Pediatrics and Intensive Care, Fundación Cardioinfantil-Instituto de Cardiología, Universidad de La Sabana, Graduate school, Universidad CES, Campus Universitario del Puente del Común, Km 7 Autopista Norte, Chía, Cundinamarca – Colombia-South America, Bogotá, Colombia; 3 Department of Pediatrics and Intensive Care, Fundación Cardioinfantil-Instituto de Cardiología, Universidad del Rosario, Bogotá, Colombia; 4 Medical School Research Department, Universidad de La Sabana, Bogotá, Colombia

**Keywords:** Viral infection, Children, Respiratory syncytial virus, Pneumonia

## Abstract

**Background:**

Acute respiratory infections (ARIs) are one of the main causes of morbidity and mortality in children. Viruses are the main etiological agents, and their behavior tends to be seasonal and vary by geographical location. Human metapneumovirus (HMPV) has recently been described as a cause of severe acute respiratory infection and its prevalence and clinical behavior in children at moderate altitudes is unknown.

**Methods:**

A cross-sectional study was carried out on patients seen at a university hospital in Bogotá, Colombia between October 2015 and December 2017 in a city at a moderate altitude above sea level. Children with acute respiratory infections who had had a multiplex RT-PCR assay were selected. The prevalence of HMPV infection, its clinical outcomes and its relationship to rainfall were evaluated.

**Results:**

Out of a total of 14,760 discharged patients, multiplex RT-PCR was performed on 502 and a virus was detected in 420 children with acute respiratory infection (ARI). The study group had a median age of 21 months (IQR 7–60), with similar proportion of males and females (56.4 and 43.6% respectively) and 5.2% (CI 95 3.3–7.8%) prevalence of HMPV infection. The group with HMPV infection showed a greater frequency of viral coinfection (22.7% vs 14% *P* = 0.03) compared with ARI caused by other viruses. The rate of bacterial coinfection (*P* = 0.31), presence of comorbidities (*p* = 0.75), length of hospital stay (*P* = 0.42), need for mechanical ventilation (*P* = 0.75) and mortality (*P* = 0.22) were similar for HMPV and other viral infections. A moderate correlation was established between HMPV infection and rainfall peaks (Spearman’s Rho 0.44 *p* = 0.02).

**Conclusions:**

Human metapneumovirus was the fifth most frequently isolated virus in children with ARI, had similar clinical behavior and severity to other viruses but a higher rate of viral coinfection. Its peaks seem to correlate to rainy seasons.

## Background

Acute respiratory infections (ARIs) are one of the main contributors to the burden of disease in pediatrics. It is estimated that children under 5 years of age have an average of three to six episodes of ARI per year, with ARI being the second cause of death in this age group according to the World Health Organization (WHO) [1]. Viruses are the most commonly isolated microorganisms in acute respiratory infection, both in adults and in children. Every year in the United States, between 20,000 to 58,000 children under 5 years of age are hospitalized due to respiratory syncytial virus (RSV) and influenza virus infections [2].

Discovered in 2001 by Dr. Van Den Hoogen, human metapneumovirus (HMPV), belonging to the *Paramyxoviridae* family, has circulated for more than five decades [3], but its importance as an etiologic agent of upper and lower ARI, with the potential for developing severe disease is emergent.

HMPV infection is responsible for approximately 4–16% of ARI hospitalizations in pediatrics [4], of those affected 15–25% needs transfer to intensive care and 8–17% requires mechanical ventilation [5].

During 2007–2013 in the United States, Carly et al. estimated that each pediatric patient hospitalized for acute respiratory infection due to HMPV may spend an average of 6000 dollars per hospitalization, which is much greater than the cost of other viruses and other chronic conditions [2].

It has been suggested that the prevalence of HMPV has seasonal and geographical variations [6]. Colombia, located near the Equator, is a tropical country with five natural regions; each region maintains an average temperature throughout the year so the rainfall is the most important climatic variable that affects viral circulation. Bogotá is located in the Andean region, is one of the coldest cities in the country, located at an altitude of 2630 m above sea level, acute respiratory infections have been thought to have a different behavior at higher altitudes in terms of higher frequency and severity. Our objective is to estimate the prevalence and describe the clinical behavior of ARI caused by HMPV in pediatric patients hospitalized in a fourth level hospital in Bogotá and evaluate its association with the rainfall variations.

## Methods

A cross-sectional study was performed on children under 18 years of age hospitalized at the Fundación Cardioinfantil-IC, a tertiary university hospital located in Bogotá, Colombia. A total of 14,760 patients were discharged between October 2015 and December 2017, out of whom 502 children had a multiplex RT-PCR (FilmArray® BioMériux). This city is located 2630 m above sea level with the characteristic weather and rains patterns of tropical countries and the Andes mountains. It is characterized by an annual mean temperature of 14 °C with variations between 6 and 19 °C, and peaks of rain from March to May each year, as well as from October to November. This study was approved by the institutional ethics committee (Protocol: PM- 1090-2018).

The data was taken from the institutional electronic charts of children who were hospitalized for acute respiratory infections and who received multiplex RT-PCR (FilmArray® BioMériux), the analyte used in this technique for HMPV detection was Type 16, A1 IA10–2003 Zeptometrix 0810161CF. Patients who had had this test without experiencing respiratory symptoms and those who had been out of the city in the 2 weeks prior to hospitalization were excluded.

The respiratory multiplex RT-PCR assay (FilmArray® BioMériux) was ordered for patients with upper and lower respiratory symptoms at the attending physician’s discretion, and samples were taken from nasopharyngeal aspirates by the respiratory therapy specialist and processed within 30 min of collection, which is an institutional standard for sample handling. The RT-PCR detects 17 viruses and 3 bacteria and is currently considered to be the gold standard for detecting these microorganisms.

Clinical behavior was assessed in terms of the main complications previously described for HMPV, such as length of hospital stay, need for transfer to intensive care, need for ventilatory support and duration of mechanical ventilation. Acute respiratory infections were classified as rhinopharyngitis, laryngitis, croup, bronchiolitis, tracheitis, pneumonia or acute respiratory distress syndrome (ARDS), according to the criteria of the attending physician.

Severe ARI was defined as the need for oxygen with a fraction of inspired oxygen (FiO_2_) greater than 40%; the need for non-invasive mechanical ventilation, high flow nasal cannula ventilation or invasive mechanical ventilation; and/or hemodynamic instability requiring vasoactive support. Patients in whom HMPV was identified 48 h after admission, and who did not have respiratory symptoms on admission, were considered to have a nosocomial infection. Viral coinfection was established if two or more respiratory viruses were isolated on the RT-PCR assay, and bacterial coinfection was determined if blood cultures and/or orotracheal secretion cultures were positive and /or procalcitonin was > 0.5 μg/L.

xAdditionally, the IDEAM (Institute of Hydrology, Meteorology and Environmental Studies) database was used. This is the governmental institution responsible for the analysis and detection of climate and meteorological changes in Colombia. Monthly rainfall patterns for the described study period were analyzed looking for an association with a higher or lower frequency of acute viral respiratory infections.

### Statistical analysis

Statistical analyses were performed using STATA 14.0 (StataCorp LP, College Station, TX), using descriptive statistics for the demographic variables and central tendency and dispersion measure for continuous variables, according to the distribution of the variable defined by the Shapiro Wilk normality test. Absolute and relative frequencies were described for qualitative variables. The prevalence was estimated for the described observation period.

For factors related to severity, a bivariate analysis was performed using Student’s t-test for independent samples, when the variable was quantitative and parametric. Otherwise, a Wilcoxon rank-sum test was used. For categorical variables, tests of independence were performed using Chi-Square. If this was not possible, due to the number of observations, Fischer’s exact test was performed.

For seasonality analysis, the monthly number of infections due to human metapneumovirus was calculated, as well as the number of respiratory syncytial virus and rhinovirus/enterovirus infections, using a Spearman’s correlation analysis for non-parametric variables.

## Results

Out of a total of 14,560 pediatric patients seen during the research period, 502 multiplex RT-PCRs were performed on hospitalized patients. Altogether, 82 were excluded because the test had been performed on a patient without a diagnosis of acute respiratory infection or the patient had traveled outside of Bogotá during the 2 weeks prior to being hospitalized.

A total of 420 patients were included; the median age in the analyzed group was 43 months (IQR 9–101) in the HMPV positive group and 21 (IQR 6–57) in HMPV negative group (*P = 0.08*). The proportion of affected girls and boys was similar between groups (*P = 0.51*) (Table 1).

**Table 1:** Clinical characteristics of human metapneumovirus infection in hospitalized children

Characterístic	HMPV positive group (*n* = 22)	HMPV negative group (*n* = 398)	Total (*n* = 420)	*P-value across groups*
Age (months)	Median: 43 (IQR 9–101)	Median: 21 (IQR 6–57)	Median: 21 (IQR: 7–60)	*0.08*
Sex				*0.51*
Male	12 (54.6%)	225 (56.5%)	237 (56.4%)	
Female	10 (45.4%)	173 (43.5%)	183 (43.6%)	
Comorbidities				
Kidney disease	4 (18.2%)	36 (9%)	40 (9.5%)	*0.15*
Liver disease	3 (13.6%)	50 (12.6%)	53 (12.6%)	*0.75*
Heart disease	1 (4.5%)	67 (16.8%)	68 (16.2%)	*0.23*
Transplant	2 (9.1%)	45 (11.3%)	47 (11.2%)	*1.0*
Prematurity	3 (13.6%)	52 (13.1%)	55 (13.1%)	*1.0*
Bronchopulmonary dysplasia	2 (9.1%)	50 (12.6%)	52 (12.4%)	*0.86*
Metabolic diseases	1 (4.5%)	26 (6.5%)	27 (6.4%)	*1.0*
Primary immunodeficiency	1 (4.5%)	39 (9.8%)	40 (9.5%)	*0.84*
Cancer	4 (18.2%)	46 (11.6%)	50 (11.9%)	*0.32*
Any comorbidity	15 (68.2%)	237 (59.5%)	252 (60%)	*0.28*
Nutritional status				*0.97*
Severe malnutrition	4 (18.2%)	65 (13.3%)	69 (16.4%)	
Malnutrition	4 (18.2%)	78 (19.6%)	82 (19.5%)	
Normal	13 (59.1%)	244 (61.3%)	257 (61.2%)	
Overweight	1 (4.5%)	10 (2.7%)	11 (2.6%)	
Obesity	0 (0%)	1 (0.3%)	1 (0.3%)	
Diagnoses				*0.75*
Croup	0 (0%)	7 (1.8%)	7 (1.7%)	
Bronchiolitis	3 (13.6%)	74 (18.6%)	77 (18.3%)	
Pneumonia	10 (45.5%)	200 (50.3%)	210 (50%)	
Asthma attack	0 (0%)	18 (4.5%)	18 (4.3%)	
Recurrent wheezing	2 (9.1%)	14 (3.5%)	16 (3.8%)	
Laryngitis	0 (0%)	4 (1%)	4 (0.9%)	
ARDS	3 (13.6%)	24 (6%)	27 (6.4%)	
Rhinopharyngitis	4 (18.2%)	55 (13.8%)	59 (14.1%)	
Sinusitis	0 (0%)	1 (0.3%)	1 (0.3%)	
Tracheitis	0 (0%)	1 (0.3%)	1 (0.3%)	
Type of infection				*0.43*
HAI	5 (22.7%)	122 (30.6%)	127 (30.2%)	
Community	17 (77.3%)	276 (69.4%)	293 (69.8%)	
Bacterial coinfection	6 (27.3%)	151 (38%)	157 (37.4%)	*0.31*
Antibiotics				*0.5*
Yes	17 (77.3%)	281 (70.6%)	298 (71%)	
No	5 (22.7%)	117 (29.4%)	122 (29%)	
Viral coinfection	5 (22.7%)	56 (14%)	61 (14.5%)	*0.03*
Length of hospital stay	Median: 10.5 days (IQR 6–26)	Median: 12 days (IQR 6–27)	Median: 12 days (IQR: 6–27)	*0.42*

In 291 (69.3%) cases, at least one microorganism was detected, the most frequently isolated etiological agents being rhinovirus/enterovirus (30% (CI 95 25–34%)), RSV (19% (CI 95 15–23%)), parainfluenza 3 (7.4%), and adenovirus (5.7%). A 5.2% (CI 95 3.3–7.8%) HMPV infection prevalence was found (22 patients), making it the fifth most frequently isolated virus (Table 2). Within this group, in 17 out of 22 cases, HMPV was the only virus detected. Viral coinfection was documented in 22.7% of cases. The most frequent viral association was with the rhinovirus/enterovirus complex in 60% of children, followed by influenza A/H1–2009 and parainfluenza 3. In this regard, the frequency of HMPV viral coinfection is greater (22.7 vs 14% *P* = 0.03) than the viral coinfection of the other viruses detected. Bacterial coinfection was documented in 27.3% of patients with HMPV and in 38% of children with other infections.

**Table 2:** Respiratory multiplex RT-PCR isolates

Multiplex RT-PCR isolation	*n* = 420
Human rhinovirus/enterovirus	125 (30%)
Respiratory syncytial virus	80 (19%)
Parainfluenza virus 3	31 (7.4%)
Adenovirus	24 (5.7%)
Human metapneumovirus	22 (5.2%)
Mycoplasma pneumoniae	14 (3.3%)
Influenza A/H31	10 (2.3%)
Coronavirus NL63	6 (1.4%)
Parainfluenza virus 1	5 (1.2%)
Influenza A/H1–2009	5 (1.2%)
Parainfluenza virus 4	4 (1%)
Coronavirus OC43	4 (1%)
Influenza B	3 (0.7%)
Coronavirus HKU1	3 (0.7%)
Coronavirus 229E	2 (0.5%)
Parainfluenza virus 2	2 (0.5%)
Influenza A	1 (0.2%)
Chlamydia pneumoniae	1 (0.2%)
Influenza A/H1	0 (0%)
Bordetella pertussis	0 (0%)
Undetected	129 (30.7%)
Coinfections	61 (14.5%)

Of the patients studied, 26.6% (*n* = 112) were previously healthy and had no chronic medical conditions. The following comorbidities were found in the entire study group: congenital heart disease (16.2%), prematurity (13.1%), liver disease (12.6%), bronchopulmonary dysplasia (12.4%), cancer (11.9%), transplant (11.2%), kidney disease (9.5%), and malnutrition (35.9%). No statistically significant differences were found between those with or without metapneumovirus infection and the presence of any of the described comorbidities (Table 1).

The most frequent diagnoses for both groups were pneumonia (50%), bronchiolitis (18.3%), and rhinopharyngitis (14.1%). Altogether, 77.3% of metapneumovirus infections were community-acquired, as well as 69.4% of other respiratory infections (Table 1).

The median hospital stay for the group with HMPV infection was 10.5 days (IQR 6–26) and for those without HMPV infection was 12 days (IQR 6–27) (*P* = 0.42). Of the patients with HMPV infection, 54.5% required admission to the intensive care unit, and in the group without this infection, the rate was 48.5% (*P* = 0.58).

The need for mechanical ventilation showed a similar behavior between both groups. Therefore, 66.7% (*n* = 8) of patients with HMPV infection required ventilatory support (58.3% invasive and 8.3% non-invasive) with a median duration of mechanical ventilation of 7 days (IQR 3–17). Similarly, 48.2% (93) of children without HMPV infection required mechanical ventilation (20.9% invasive and 2.5% non-invasive) with no significant difference in terms of need (*P* = 0.88) and duration of mechanical ventilation (6 days, IQR 3–14) between the groups (Table 3). The global mortality was 7.1% of the study sample (HMPV 13.6% vs non- HMPV 6.8%, *P =* 0.2).

**Table 3:** Clinical characteristics of human metapneumovirus infection in the intensive care unit

Characteristic	HMPV positive group (*n* = 22)	HMPV negative group (*n* = 398)	Total (*n* = 420)	*P-value across groups*
PIM 2	Median: 0.6% (IQR 0.3–1%)	Median: 0.9% (IQR 0.3–2.8%)	Median: 0.9% (IQR: 0.3–2.8%)	*0.2*
Length of PICU stay	Median: 7 days (IQR 4–14.5)	Median: 6 days (IQR 3–15)	Median: 6 days (IQR 3–15)	*0.87*
Mortality	3 (13.6%)	27 (6.8%)	30 (7.1%)	*0.22*
Type of Ventilation				*0.75*
Invasive	7 (31.8%)	83 (20.9%)	90 (21.4%)	
Non-invasive	1 (4.6%)	10 (2.5%)	11 (2.6%)	
HFNC	4 (18.2%)	70 (17.9%)	74 (17.6%)	
Nasal cannula	5 (22.7%)	134 (33.7%)	139 (33%)	
Ventury	0 (0%)	8 (2.01%)	8 (1.9%)	
None	5 (22.7%)	93 (23.4%)	98 (23.3%)	
Days on Mechanical Ventilation	Median: 7 days (IQR 3–17)	Median: 6 days (IQR 3–14)	Median: 6 days (IQR 3–17)	*0.58*

*HMPV* Human metapneumovirus, *PIM 2* Pediatric Index Mortality 2 Scale, *PICU* Pediatric intensive care unit, *HFNC* High flow nasal cannula

Out of the 22 patients with HMPV, 54% were found to have severe respiratory infection (Table 4). This group with severe HMPV, 58.3% were under 2 years old, while only 20% of those without severe HMPV infection belonged to this age group (*P = 0.27*).

**Table 4:** Severe HMPV infection

HMPV positive	PICU (*n* = 12)	NO PICU (*n* = 10)	*P*
Age			*0.27*
Under 1 year	5 (41.6%)	2 (20%)	
1–2 years	2 (16.7%)	0 (0%)	
2–5 years	2 (16.7%)	4 (40%)	
Over 5 years	3 (25%)	4 (40%)	
Sex			*0.39*
Male	8 (66.7%)	4 (40%)	
Female	4 (33.3%)	6 (60%)	
Comorbidities			
None	2 (16.7%)	2 (20%)	*1.0*
Kidney disease	2 (16.7%)	2 (20%)	*1.0*
Liver disease	2 (16.7%)	1 (10%)	*1.0*
Heart disease	1 (8.3%)	0 (0%)	*0.2*
Transplant	0 (0%)	2 (20%)	*0.2*
Prematurity	3 (25%)	0 (0%)	*0.48*
Bronchopulmonary dysplasia	2 (16.7%)	0 (0%)	*1.0*
Metabolic disease	1 (8.3%)	0 (0%)	*1.0*
Primary immunodeficiency	1 (8.3%)	0 (0%)	*0.03*
Cancer	0 (0%)	4 (40%)	
Nutritional status			*0.59*
Severe malnutrition	3 (25%)	1 (10%)	
Malnutrition	2 (16.7%)	2 (20%)	
Normal	6 (50%)	7 (70%)	
Overweight	1 (8.3%)	0 (0%)	
Obesity	0 (0%)	0 (0%)	
Diagnoses			*0.07*
Pneumonia	7 (58.3%)	3 (30%)	
ARDS	3 (25%)	0 (0%)	
Bronchiolitis	1 (8.3%)	2 (20%)	
Recurrent wheezing	1 (8.3%)	1 (10%)	
Rhinopharyngitis	0 (0%)	4 (40%)	
Viral coinfection	4 (33.3%)	1 (10%)	*0.32*
Bacterial coinfection	6 (50%)	1 (10%)	*0.16*

Viral coinfection was more frequent in the group with severe HMPV infection (*n* = 12) (33% vs 10%, *P* = 0.32), compared to those who did not have it. Likewise, the bacterial coinfection rate was considerably greater in patients with severe infection (50%) compared to those who did not have severe infection (10%) (*P* = 0.16).

**Fig. 1:**
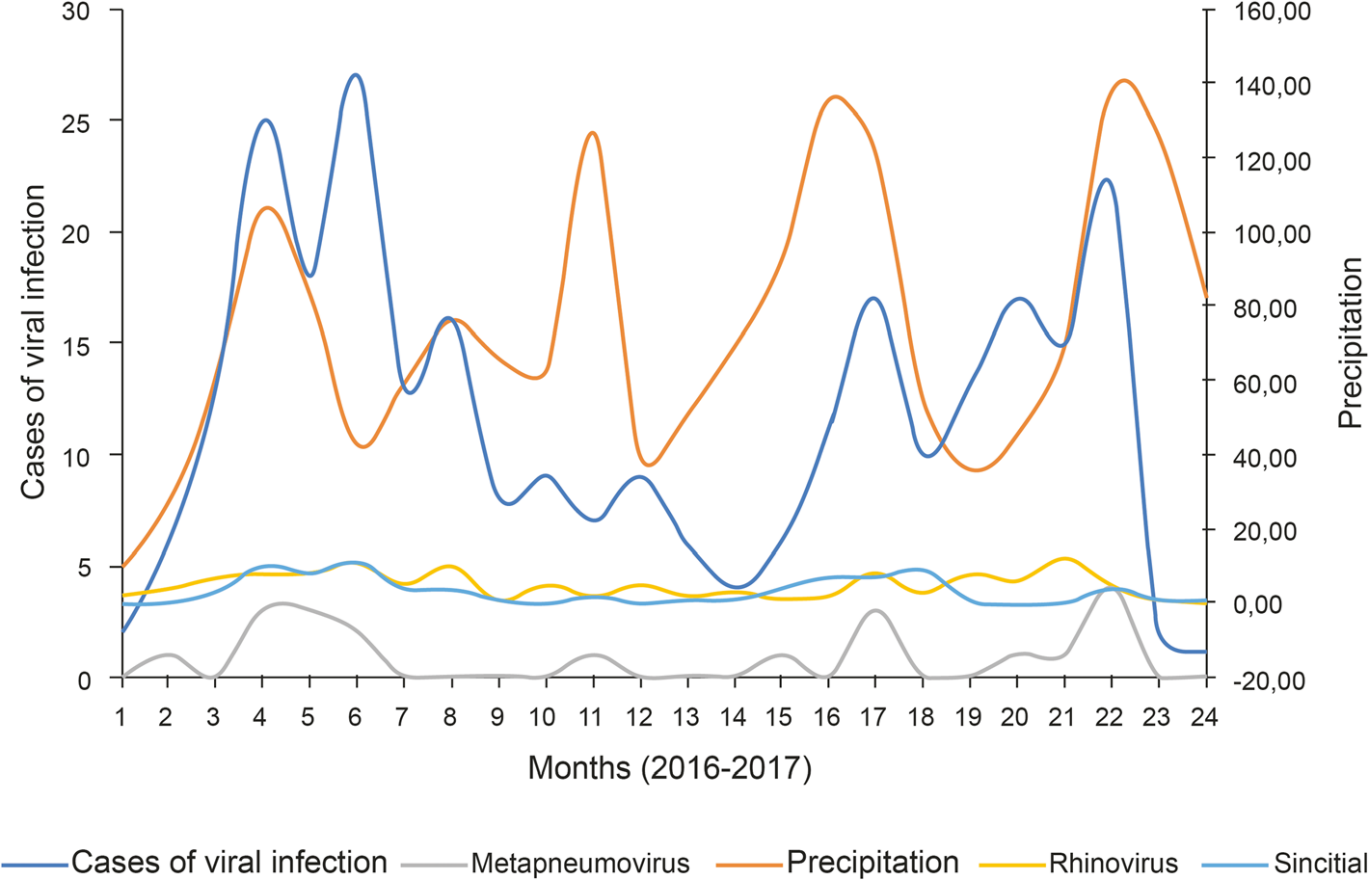
Distribution of human metapneumovirus, rhinovirus and respiratory syncytial infections as related to rainfall during 2016–2017

**Fig. 2:**
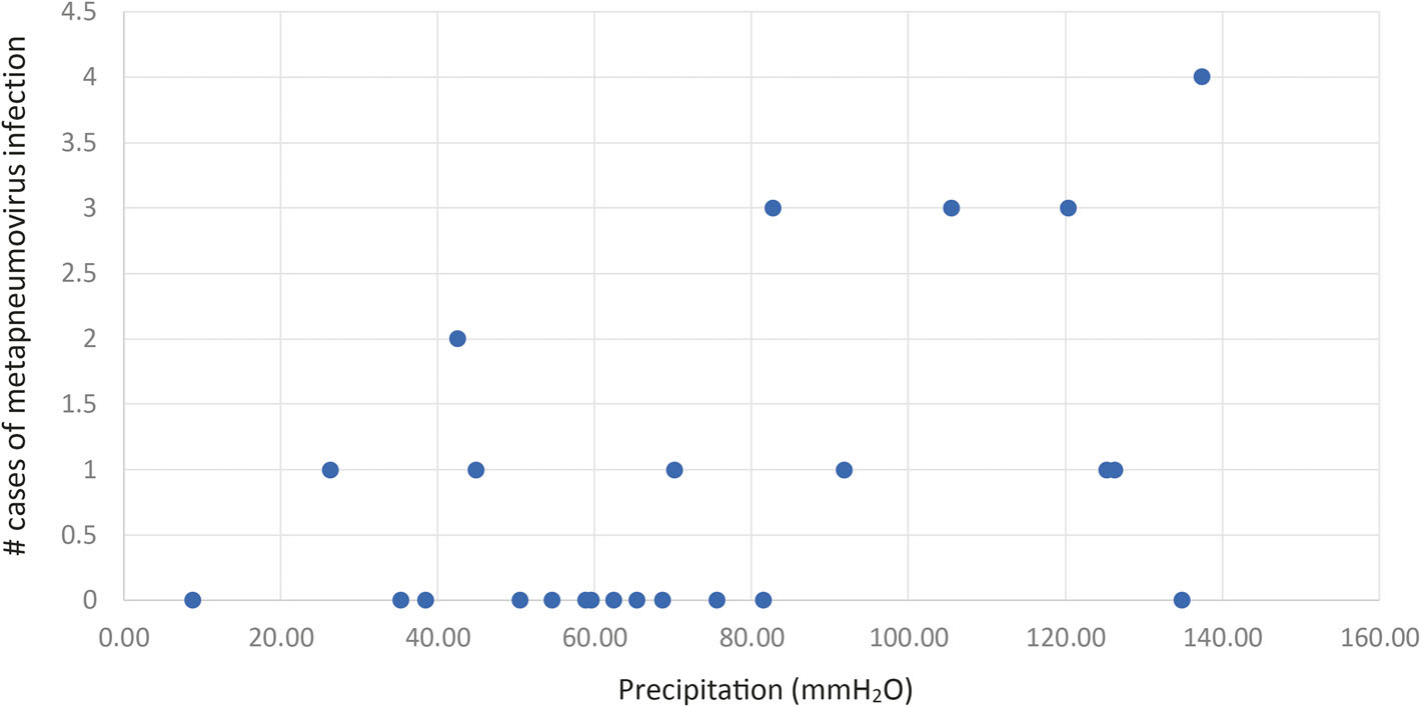
Dispersion diagram of the Metapneumovirus infection and rainfall peaks

The rainfall in the city of Bogotá was analyzed using the average rainfall reported by the 14 IDEAM (Institute of Hydrology, Meteorology and Environmental Studies) monitoring stations (Fig. 1). Typical tropical zone behavior was observed with a greater frequency of viral infections during the rainy season. The Spearman Rho correlation analysis showed a moderate correlation (*r* = 0.44, *P* < 0.02) between the presence of HMPV infection and the highest rainfall peaks, just as occurs with RSV (*r* = 0.32, *P* < 0.02) (Fig. 2). However, this correlation was not seen with the rhinovirus/enterovirus complex, which was the most frequently detected co-infecting agent for HMPV (*r* = − 0.13).

## Discussion

In this study, we investigated the prevalence of HMPV in pediatric patients hospitalized in Bogota, Colombia. We found a 5.2% prevalence of HMPV infection (CI 95% 3–3 - 7.8%). It was the fifth virus detected in hospitalized patients. In Latin America, the prevalence in hospitalized patients is highly variable, being reported as high as 20% in the southern Brazil [7], 11% in Argentina [8] and 9.2% in Uruguay [9]. This behavior is most likely explained by the variable climate changes between the Latin American countries and the techniques used for viral detection [10].

HMPV has often been described as a coinfectant with other viruses. In children, viral coinfection is highly variable for HMPV [11]. In Spain, there was an estimated rate of 38% [12], in Jordan it was 52.5% [13], and in south China it was 18.4% [14]. The viral coinfection rate has not been described for HMPV in children in Colombia, which in our study was 14%. However, we found a greater frequency of viral coinfection in the HMPV group compared with coinfection of the other respiratory viruses, and this difference was statistically significant (22.7% vs 14%, *P* = 0.03).

The most frequently described viral association in respiratory infection has been between RSV and HMPV. Bear in mind that they belong to the same viral family and have a high genetic similarity, usually presented during the same epidemiological periods. In a recent article, Schuster et al. found that RSV was the main coinfecting agent, with a global rate of coinfection of 26.4% [13]. In our study, rhinovirus/enterovirus was the main agent presenting simultaneously with HMPV in 60% of cases. Contrary to the findings of this author, we did not find RSV coinfection in these children.

With respect to bacterial coinfection, García-García et al. reported that 25.6% of the patients in their study received concomitant antibiotic treatment due to suspected bacterial infection associated with HMPV infection [12], but the frequency of presentation is not documented. In our study, we found that 27.3% of patients positive for HMPV had bacterial coinfection documented by PCR, cultures or positive procalcitonin techniques.

Regarding the clinical behavior of HMPV infection, it has been reported that 2.2–7% of affected patients require admission to intensive care [12, 15]. In our study, the rate of transfer of those infected with HMPV and not infected with HMPV was much higher (54.5% (*n* = 12) and 48.5% (*n* = 198), respectively) than that described by Edwars et al. and García-Garcia et al. with a transfer ICU of 2.8 and 6% respectively [12, 15]. Likewise, the length of stay in the ICU for those infected with HMPV according to the study by Edwards et al. was 4.5 days. In our study group it was 7 days (IQR 4–14.5). It is possible that our population could have different serotypes, which would explain this behavior. Another possibility is that living at a higher altitude above sea level might at least partially explain a more aggressive presentation of these infections resulting in more patients being transferred to intensive care or remaining hospitalized for longer periods. Studies are needed to compare the clinical behavior of children with acute respiratory infections living at sea level with that of children living at moderate or high altitudes.

Of the patients hospitalized in intensive care, we observed that 31.8% of children infected with HMPV required invasive mechanical ventilation and 4.6% non-invasive with ventilation lasting for 7 days on average. This is similar to what was observed by Lozano et al. in Chile [16], they found that the duration of ventilatory support in patients with HMPV was 7 days and that 25% of their children required invasive ventilatory support. We found that children who required a PICU stay had pneumonia more often than children with HMPV who did not need intensive care (58.3% vs 30% respectively), which could partially explain these findings.

Inpatient outbreaks of RSV in children have been described on hospital floors and in intensive care units [17], but inpatient HMPV outbreaks are rarely reported [18]. In this study, one out of five children acquired the HMPV infection during their hospital stay. Nosocomial HMPV infections with molecular confirmation have been described in cancer patients, and outbreaks have also been described in geriatric institutions and on psychiatric wards [19, 20]. Contact seems to be required for viral transmission; the analysis of this situation forces us to look for strategies to decrease inpatient viral transmission by improving our isolation conditions and patient management [21]. More studies are needed to determine the risk factors for acquiring nosocomial HMPV infection.

Considering that Bogotá is located in a zone without seasons, it does not have the drastic temperature changes like other countries. The most important climatic variable that can affect viral circulation is rainfall. It has been postulated that viral transmission increases in colder seasons. Also, that the antiviral immune response in the nasal epithelium is attenuated under these circumstances [21, 22].

Previous studies have shown that HMPV may have alternating epidemiological profiles in Europe. It has been found that the percentage of annual HMPV infection may vary year to year, and may oscillate between 2.3 and 19.9% in the same region [12, 23]. The annual prevalence difference in our study was 4.4% in 2016 and 5.6% in 2017. A greater frequency was noted in April–May 2016, April–June and September–October 2017, with a low positive correlation between HMPV infection and the highest rainfall peaks (*r* = 0.44, *P* < 0.02).

The differences in this circulation pattern, besides being related to changes in rainfall, may be associated with the circulation of different HMPV serotypes [24]. Four different HMPV lineages are known, designated as A1, A2, B1 and B2. These may coexist in the same period or one may be predominant [25]. The respiratory RT-PCR used in this study does not allow for serotype differentiation, and therefore no conclusion can be drawn regarding their differential behavior.

Our study has several limitations. The findings are from a single tertiary care center, and although multiplex RT-PCR was used, it was not performed on all hospitalized patients with acute respiratory infections due limitations of health system. Likewise, this test does not identify serotypes (A1, A2, B1, B2) which could partially explain some different seasonal behaviors of the virus. Similarly, the study design allowed us to observe the behavior of the virus over a specific time period; this behavior may change according to serotypes and environmental variations, among others. Additionally, our data only represents association because we cannot infer that HMPV was the only causal factor of the observed disease, especially in cases with viral coinfection.

## Conclusion

Human metapneumovirus was the fifth most commonly detected acute respiratory infection virus in this study. Its clinical behavior at moderate altitudes in terms of severity is similar to that of other respiratory viruses. These children frequently need to be transferred to intensive care and have viral coinfection. We observed that this infection presents more frequently during the rainy season.

## Data Availability

Datasets used and/or analyzed during the current study are available from the corresponding author on reasonable request.

## Abbreviations

ARDS: Acute respiratory distress syndrome
ARI: Acute respiratory infections
FiO_2_: Fraction of inspired oxygen
HMPV: Human metapneumovirus
IDEAM: Institute of Hydrology, Meteorology and Environmental Studies
RSV: Respiratory syncytial virus
RT-PCR: Real time-Polymerase chain reaction
WHO: World Health Organization
